# Structural and functional insights into the periplasmic detector domain of the GacS histidine kinase controlling biofilm formation in *Pseudomonas aeruginosa*

**DOI:** 10.1038/s41598-017-11361-3

**Published:** 2017-09-12

**Authors:** Ahmad Ali-Ahmad, Firas Fadel, Corinne Sebban-Kreuzer, Moly Ba, Gauthier Dangla Pélissier, Olivier Bornet, Françoise Guerlesquin, Yves Bourne, Christophe Bordi, Florence Vincent

**Affiliations:** 1 CNRS, Aix Marseille Univ, AFMB, Marseille, France; 2 LISM, IMM, Aix-Marseille Univ and CNRS, Marseille, 13402 France

## Abstract

*Pseudomonas aeruginosa* is an opportunistic pathogenic bacterium responsible for both acute and chronic infections and has developed resistance mechanisms due to its ability to promote biofilm formation and evade host adaptive immune responses. Here, we investigate the functional role of the periplasmic detector domain (GacS_PD_) from the membrane-bound GacS histidine kinase, which is one of the key players for biofilm formation and coordination of bacterial lifestyles. A *gacS* mutant devoid of the periplasmic detector domain is severely defective in biofilm formation. Functional assays indicate that this effect is accompanied by concomitant changes in the expression of the two RsmY/Z small RNAs that control activation of GacA-regulated genes. The solution NMR structure of GacS_PD_ reveals a distinct PDC/PAS α/β fold characterized by a three-stranded β-sheet flanked by α-helices and an atypical major loop. Point mutations in a putative ligand binding pocket lined by positively-charged residues originating primarily from the major loop impaired biofilm formation. These results demonstrate the functional role of GacS_PD_, evidence critical residues involved in GacS/GacA signal transduction system that regulates biofilm formation, and document the evolutionary diversity of the PDC/PAS domain fold in bacteria.

## Introduction

To cope with environmental changes and develop colonization strategies, bacteria have evolved several sensing systems, including cell-surface signaling systems, quorum sensing, cyclic di-GMP, and the predominant two-component signal-transduction systems (TCS). By modulating cellular functions in response to environmental changes, TCSs play essential roles for the adaptation and survival of organisms^1, 2^. Typically, a TCS comprises a membrane-embedded histidine kinase sensor (HK), which acts mainly as a dimeric assembly, and a cognate response regulator (RR). Detection of an environmental stimuli by the HK detector domain triggers autophosphorylation of the HK cytoplasmic domain^3, 4^, leading to activation of a phosphorelay mechanism ending onto the cognate RR to mediate expression of various target genes^5^.

*P. aeruginosa* is a major opportunistic pathogen, responsible for nosocomial infections causing severe infections in vulnerable patients such as those with cystic fibrosis or hospitalized with cancer, severe burns and in intensive care units. *P. aeruginosa* is able to switch from a planktonic (free swimming) to a sessile (biofilm) lifestyle and several TCSs play a critical role in controlling this switch^6, 7^. In the free-swimming state responsible for acute infection, bacteria can cross host barriers and proliferate inside the host using motility and virulence factors that are secreted in the extracellular space or directly injected into the host cells using the type III secretion systems^8^. Chronic infection is characterized by formation of an antibiotic-resistant biofilm in which intricate bacterial communities are embedded within a matrix of exopolysaccharides and DNA^9^. In this particular state, bacteria concomitantly secrete toxins delivered by the type VI secretion system (H1-T6SS), which are used to kill and compete with other species in a crowded and enclosed community^10–13^.

Many reports have described a balance between expression of molecular determinants involved in chronic infection (biofilm) and those involved in acute infection (cytotoxicity). In *P. aeruginosa*, the HK/RR pair made by the GacS/GacA TCS, which plays a central role for controlling the transition state between the two infection types, is antagonistically modulated by three other histidine kinase sensors LadS, RetS, and PA1611^14, 15^. The calcium-responsive LadS HK activates GacA by using GacS as a direct phosphorelay mechanism to promote chronic infection^16, 17^. Conversely, the RetS HK blocks GacA activation by impeding GacS autophosphorylation to promote acute infection^18^. In this regulatory scheme, the PA1611 HK permits GacS activation by preventing the interfering effects of the RetS HK on the GacS signalling pathway^19, 20^.

Within the HK family, activation of this phosphorylation cascade requires recognition of an external signal molecule by a highly variable detector domain, which can be embedded in the cytoplasmic, the inner membrane or the periplasmic space^3, 4^. The nature of the signal sensed by the HKs are broad and can include nutriments, ions, temperature or redox state^21^. During the last decade, several detector domains have been characterized and various structural families have been proposed despite sequence discrepancy. In turn, three large families of detector domains have been defined according to sequence similarity and fold: 1- an α-helical fold like the *E. coli* NarX detector domain, 2- a β-sheet fold like the *P. aeruginosa* RetS periplasmic detector domain and 3- a mixed α/β fold named PAS-like/PhoQ, DcuS and CitA (PDC) domain^3, 22–24^.

The GacS HK harbors a N-terminal transmembrane α-helix, followed by a periplasmic detector domain (GacS_PD_) tailed by a second transmembrane α-helix connected to a large cytoplasmic region^25^. Unlike classical HKs made of a single cytoplasmic transmitter domain (H1), the unorthodox GacS HK consists of a transmitter domain (H1) linked to the two phosphotransfer receiver (D1) and transmitter (H2) domains. Once activated, the GacA RR positively and exclusively controls expression of two unique target genes encoding the two small noncoding RsmY and RsmZ RNAs^26^. These two RsmY and RsmZ RNAs sequesters the small RNA-binding protein RsmA, a translational repressor of genes regulating biofilm, such as the polysaccharide *pel* and *psl* ^27, 28^, the type VI secretion system (H1-T6SS) and associated virulence factors, or cytotoxicity such as the type III secretion system (T3SS)^29, 30^.

The GacS HK possesses a periplasmic 126-residue detector domain which is proposed to recognize a yet unknown signal and transmit structural rearrangements onto the transmembrane helices, leading to activation of the phosphorelay cascade^25^. While the series of events occurring in this regulatory mechanism have been well documented at the molecular level, as exemplified by the calcium-responsive LadS HK^17^, the architecture of GacS_PD_ and the molecular determinants underlying signal response remain to be investigated. Here, we report the functional and structural characterization of *P. aeruginosa* GacS_PD_. We show that a *P. aeruginosa* mutant strain lacking GacS_PD_ exhibits altered biofilm formation and *rsm* gene expression. The solution nuclear magnetic resonance (NMR) structure of GacS_PD_ reveals an atypical PDC/PAS-like domain fold that consists of a 3-stranded β-sheet flanked by 3 α-helices on one face and a major loop region on the opposite face. Mapping of conserved surface-exposed residues identifies a putative functional pocket that could act as a ligand-binding site created primarily by residues from the major loop. NMR relaxation experiments indicate that this major loop is conformationally dynamic in solution, suggesting that ligand-induced conformational changes may occur. Mutation of three residues lining this putative binding pocket causes severe defects in biofilm formation and *rsm* genes expression, suggesting a functional role of these residues in the downstream GacS/GacA signal transduction system. Overall, these results unveil the functional role of GacS_PD_ and document the evolutionary diversity of PDC/PAS domain fold in bacteria. They provide new insights into the central role of the *P. aeruginosa* GacS/GacA TCS to control bacterial lifestyle through the GacS-mediated signaling transduction mechanism.

## Results and Discussion

### The GacS periplasmic detector domain is required for GacS function

**Figure 1:**
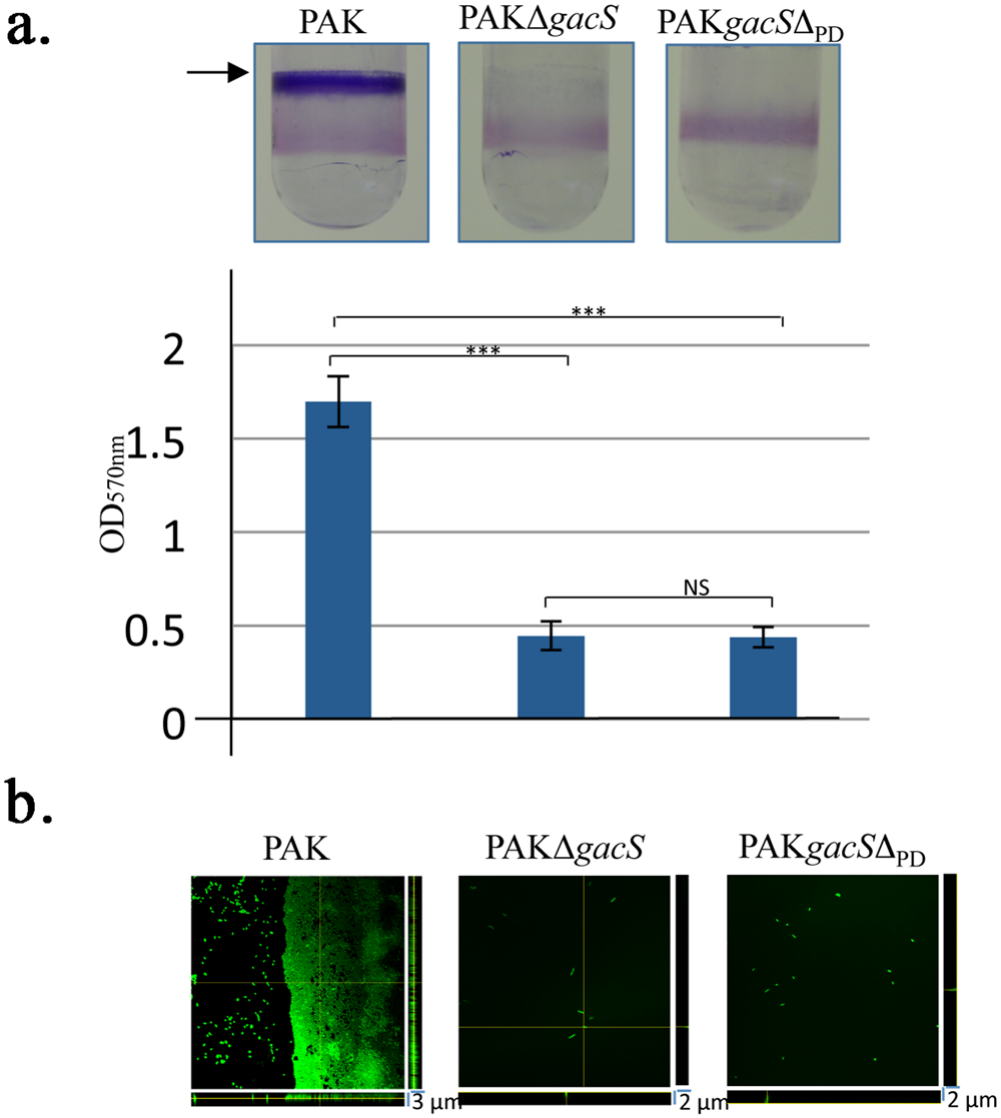
Deletion of the GacS periplasmic detection domain affects biofilm formation. (**a**) Biofilm production in glass tubes (upper panel) is illustrated and quantified after crystal violet-staining (lower panel). Biofilm levels represent mean values (with error bars) obtained from three independent experiments. *, **, *** and ns refer to p < 0.05, p < 0.01, p < 0.001 and non-significant difference, respectively, according to the Wilcoxon-Mann-Whitney tests. (**b**) Biofilm formation is monitored by confocal laser scanning microscopy after 12 h. The extracted z images and their respective xy and xz planes are shown.

**Figure 2:**
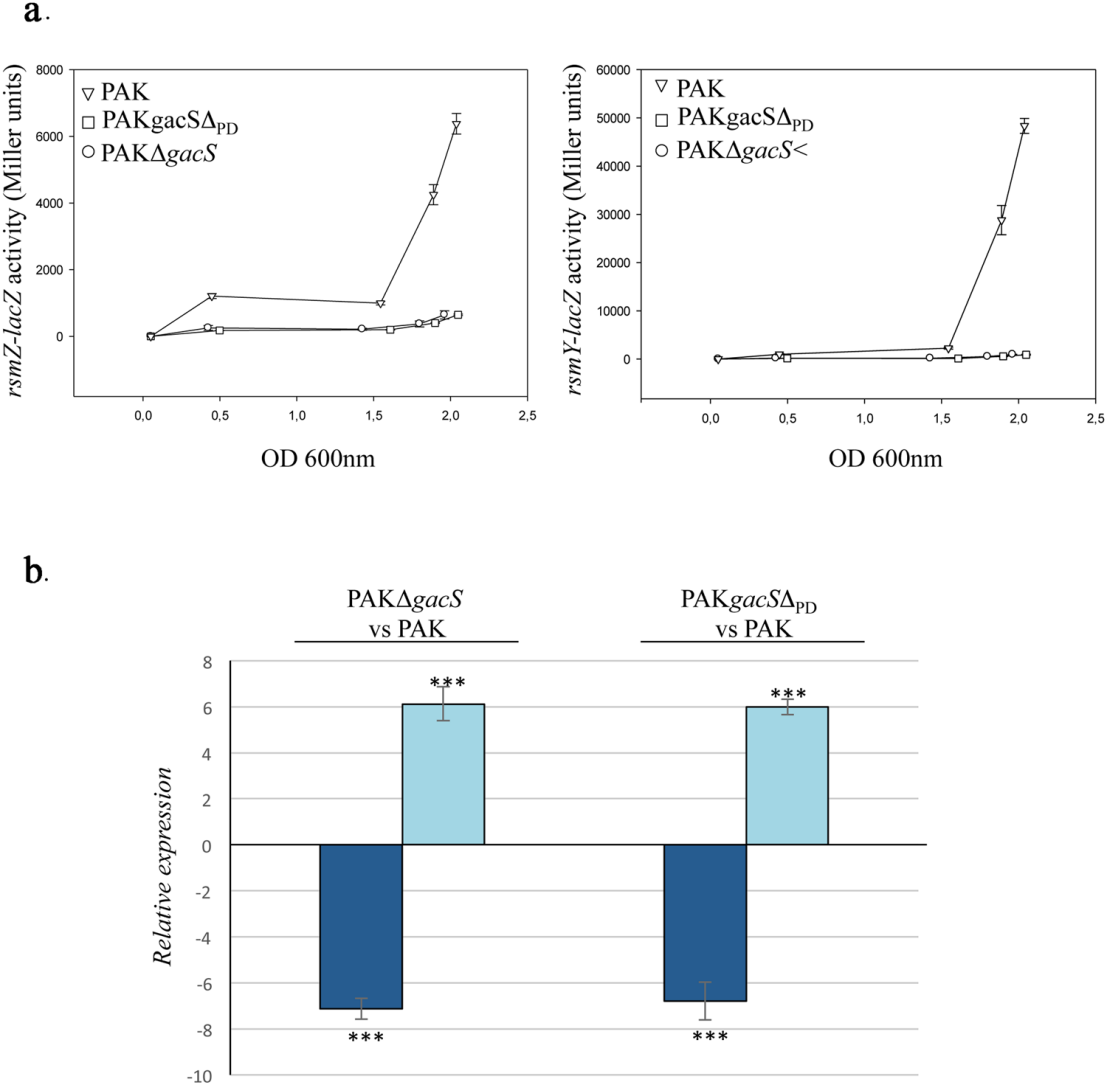
Effect of the deletion of the GacS detection domain on *rsm*, T3SS and T6SS gene expression. (**a**) Activities of the *rsmZ–lacZ* (left) and *rsmY–lacZ* (right) transcriptional chromosomal fusions were monitored at different growth stages in the PAK WT (open triangle), PAKΔ*gacS* (open square) or PAK*gacS*Δ_PD_ (open circle) strain. The corresponding β-galactosidase activities are expressed in Miller units as the mean values (with error bars) of three independent experiments. (**b**) Transcript levels of VgrG1b (T6SS; dark blue bar) and ExoS (T3SS; light blue bar) were monitored in the PAK, PAK∆*gacS* and PAK*gacS*Δ_PD_ strains using RT-qPCR; fold change is displayed for the two mutant strains compared to PAK strain. *, ** and *** refer to p < 0.05, p < 0.01 and p < 0.001, respectively, according to the moderated t-tests.

To evaluate the functional role of the GacS_PD_ domain in activation of the GacS/GacA signaling pathway in *P. aeruginosa*, we performed phenotypic analysis related to biofilm formation using the PAK*gacS*Δ_*PD*_ strain harboring a GacS HK variant lacking 102 residues, of the periplasmic detector domain (Table S1). The PAK*gacS*Δ_*PD*_ strain exhibits severe defects in biofilm formation as determined by crystal violet staining, a phenotype similar to the PAKΔ*gacS* mutant strain (Fig. 1a). To better quantify the amount of biofilm produced by the PAK*gacS*Δ_*PD*_ strain, we in-depth analyzed the biofilm morphology of the three WT, PAKΔ*gacS* and PAK*gacS*Δ_*PD*_ strains by confocal laser scanning microscopy using DAPI-labelled cells (Fig. 1b). Consistent with the crystal violet-based assay, biofilm image analysis of the PAK*gacS*Δ_*PD*_ strain evidences a lack of biofilm structure reminiscent of the PAKΔ*gacS* strain, corresponding to around 4-fold reduction in biofilm thickness compared to a compact multilayer structure of the WT PAK strain (Fig. 1b). Next, we examined whether deletion of GacS_PD_ impairs the GacS/GacA TCS signaling pathway. We thus monitored the expression profiles of the two *rsmZ* and *rsmY* genes by introducing the *rsmY–lacZ* and *rsmZ–lacZ* transcriptional fusions in the PAK*gacS*Δ_*PD*_ strain. Analysis of the level of β-galactosidase activity, measured at various growth stages, revealed a reduced *rsmY* and *rsmZ* promoter activity in the PAK*gacS*Δ_*PD*_ strain compared to the parental WT PAK strain (Fig. 2a). Hence, expression level of the two sRNAs was decreased by 61-fold and 11-fold for *rsmY* and *rsmZ*, respectively, in the *gacS*Δ_PD_ mutant PAK strain compared to the WT strain (Fig. 2a). Since up-regulation of *rsmY* and *rsmZ* genes leads to T3SS repression and T6SS up-regulation^14, 31^, we also examined whether a PAK*gacS*Δ_*PD*_ mutant strain could affect T3SS or T6SS expression. By monitoring expression level of *vgrG1b* or *exoS*, which are two specific components of T6SS and T3SS, respectively, we found a 6.1-fold induction of T3SS associated to a 7.1-fold repression of T6SS in the GacSΔ_PD_ HK variant (Fig. 2b), consistent with a similar expression level of these two components in the PAK Δ*gacS* strain.

To evidence the functionality of the GacS variant lacking the periplasmic detector domain produced by the PAK*gacS*Δ_PD_ strain, we checked the capacity of the LadS HK to activate expression of *rsm* genes. We had previously reported that the cytoplasmic region of GacS HK is required for the LadS HK to activate biofilm formation and expression of *rsmY* and *rsmZ* genes^16^. Thus, we overexpressed the full-length LadS protein using the pBBRladS plasmid in the WT, PAKΔ*gacS* and PAK*gacS*Δ_*PD*_ strains containing the *rsmY-lacZ* or *rsmZ-lacZ* fusion. Overexpression of LadS results in a significant increase of biofilm formation and activity of both *rsm* fusions in the WT and PAK*gac*SΔ_PD_ strains (Fig. S1), suggesting that a GacS variant lacking the periplasmic detector domain is responsive to LadS activation. Taken together, these data demonstrate the functional role of the GacS periplasmic dectector domain to activate the GacS/GacA signaling pathway and modulate expression of the two *rsm* genes for production of the T3SS or T6SS effectors. The fact that the GacS periplasmic domain from the root-colonizing *P. fluorescens* CHA0 strain was shown to not have essential activity for the GacS/GacA signaling pathway^32^ argues for possible different functions within a group of evolutionary-related proteins. We thus generated a phylogenic tree using 227 sequence homologues of the GacS periplasmic detector domain (Fig. S2). As evidenced by the phylogenic tree, the periplasmic domains of *P. fluorescens* and *P. aeruginosa* GacS appear to have early diverged within a *Pseudomonas* group reinforcing the hypothesis that these proteins can evolve a new or modified function. This is consistent with the recent observation that even closely related LadS orthologues are unable to sense calcium pointing to the acquisition of new functions by the periplasmic detector domains during evolution of this HK family^10^.

### The NMR solution structure of GacS_PD_ reveals a distinct PDC α/β fold domain

To decipher the architecture and functional determinants of GacS_PD_ (Met38-Gly164)_,_ a construct harboring an N-terminal TEV-cleavable His_6_-tag was expressed in *E. coli* and purified as previously reported^33^. Size-exclusion chromatography (SEC)-MALS analysis reveals a dominant monomeric population with an estimated molecular weight of 15 kDa (Fig. S3). ^15^N- and ^13^C-labelled GacS_PD_ produced in ^15^N and ^13^C-labelled minimal medium was used to achieve a complete resonance assignment of GacS_PD_ obtained using standard multidimensional triple resonance NMR experiments^33^.

The GacS_PD_ solution structure is based on 1299 non-redundant and unambiguously nuclear Overhauser effect (NOE)-derived distance restraints, 94 dihedral angle restraints and 27 hydrogen bond restraints (Table S2). The final ensemble of 20 best low-energy NMR structures has a rmsd value of 0.95 Å for the backbone (N, Cα and Co) atoms, and exhibits no obvious NOE violations and dihedral violations >0.5 Å. The rmsd value of backbone residue atoms in regular secondary structure elements is 0.35 Å for 524 atoms.

**Figure 3:**
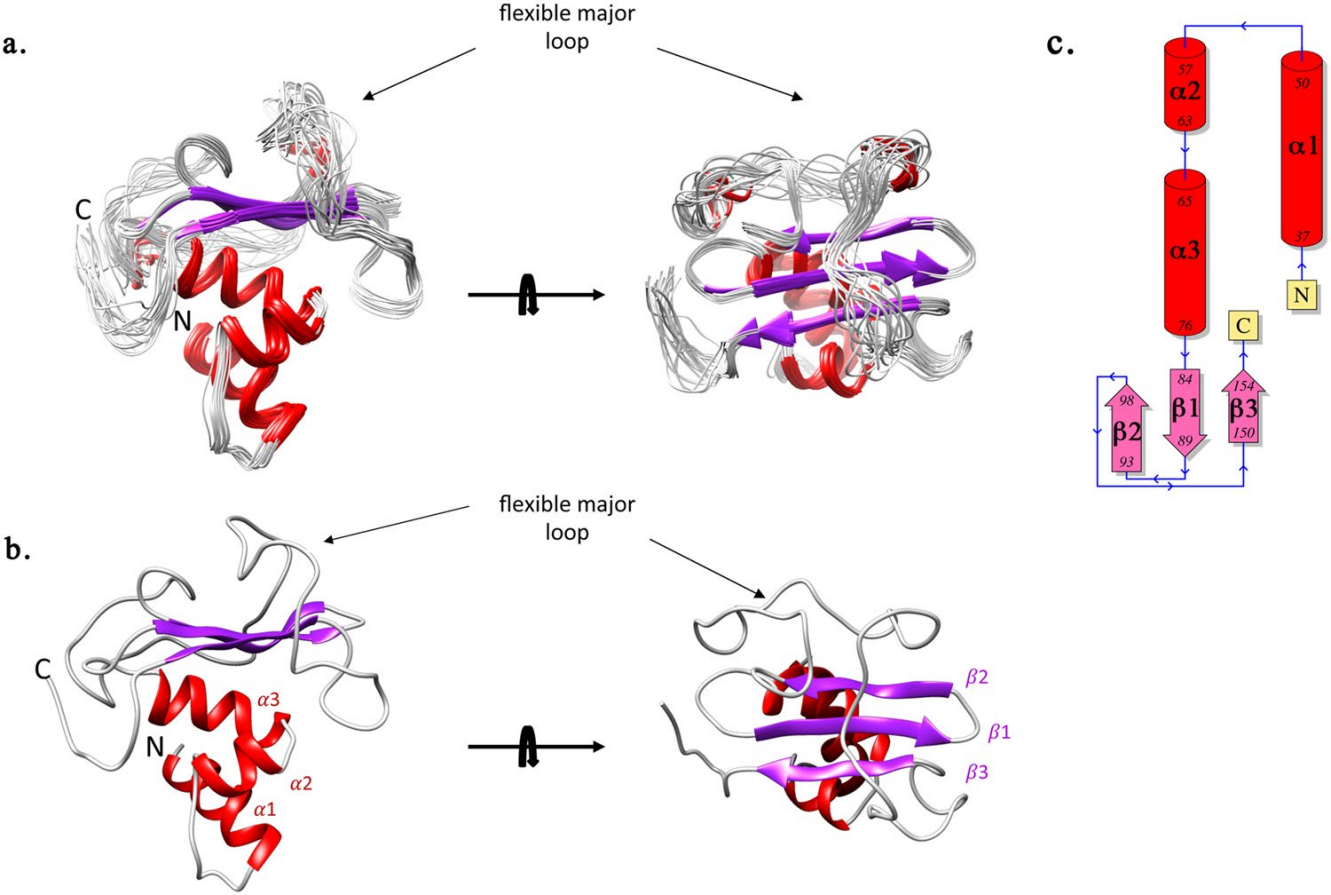
NMR solution structure of GacS_PD_. (**a**) A superimposition of 20 representative structures of GacS_PD_ corresponding to the minimal rmsd of all protein backbone N, Cα and CO atoms. (**b**) GacS_PD_ lowest-energy structure. The structure contains a central β-sheet containing three β-strands (β1 84–89, β2 93–98 and β3 150–154) and three N-terminal α-helices (α1 38–50, α2 57–63 and α3 65–76). A major loop, indicated by a black arrow, links β2 to β3 and wraps the apical side of the β-sheet. The N- and C-termini are labelled. (**c**) Topology scheme of GacS_PD_.

The GacS_PD_ solution structure consists of a central three-stranded antiparallel β-sheet flanked by three N-terminal α-helices on one side and a major loop on the opposite side. The α3-β1/2-loop-β3 topology found in GacS_PD_ is reminiscent of the α3-β2-α1/2-β3-α topology found in other extracytoplasmic PDC/PAS domains (Fig. 3). In fact, the GacS_PD_ three-stranded β-sheet differs from the extended five-stranded β sheet typically found in canonical PDC/PAS fold structures^34^ (Fig. S4). The major 49-residue length loop (Gly99-Leu148), which connects β2 to β3, wraps one face of the β-sheet and part of the outer face of helix α3 that is tightly packed against the opposite face of the β-sheet^34, 35^ (Fig. 3). Despite the sparse NOE contacts for the major loop region that mainly comprise intra-residue and sequential contacts (Fig. S5), nine long-range NOE distance restraints could be identified between residues from the loop and the β-sheet (Thr123/Gly93, Thr123/Arg94, His124/Gly93, His124/Arg94, Leu125/Gly93, Leu125/Arg94, Gly131/His97, Gly131/Thr86, Ala139/Trp150).

**Figure 4:**
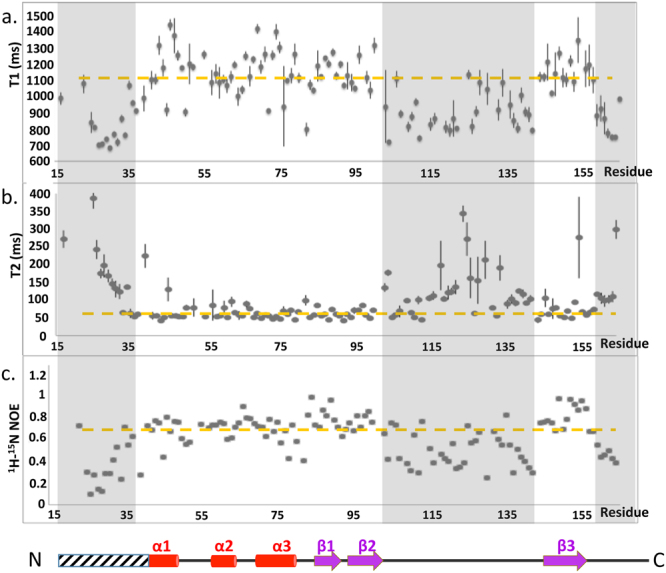
^15^N NMR backbone relaxation data of GacS_PD_. (**a**) Per residue ^15^N T_1_ longitudinal relaxation times. (**b**) Per residue ^15^N T_2_ transverse relaxation times. (**c**) Per residue heteronuclear NOE ratios along with the location of GacS_PD_ secondary structural elements shown as red cylinder for α-helix and violet arrow for β-strand.

To evaluate the dynamics behavior of GacS_PD_ and its major loop in solution, we determined the NMR relaxations properties on the pico to nanosecond timescale of the backbone amides. Most residues showed {^1^H-^15^N} NOE ratio values above 0.8 indicating highly defined structures with low flexibility (Fig. 4). In contrast, residues in the major loop (as well as in the N- and C-termini) displayed NOE ratio values below 0.4 together with shorter transversal relaxation time (T1) and longer longitudinal relaxation time (T2), which reflect higher flexibility. These results are in excellent agreement with NOE-based secondary structures and confirm the inherent flexibility of the GacS_PD_ major loop.

### Structural comparison evidences a conserved residue pattern despite fold variation

**Figure 5:**
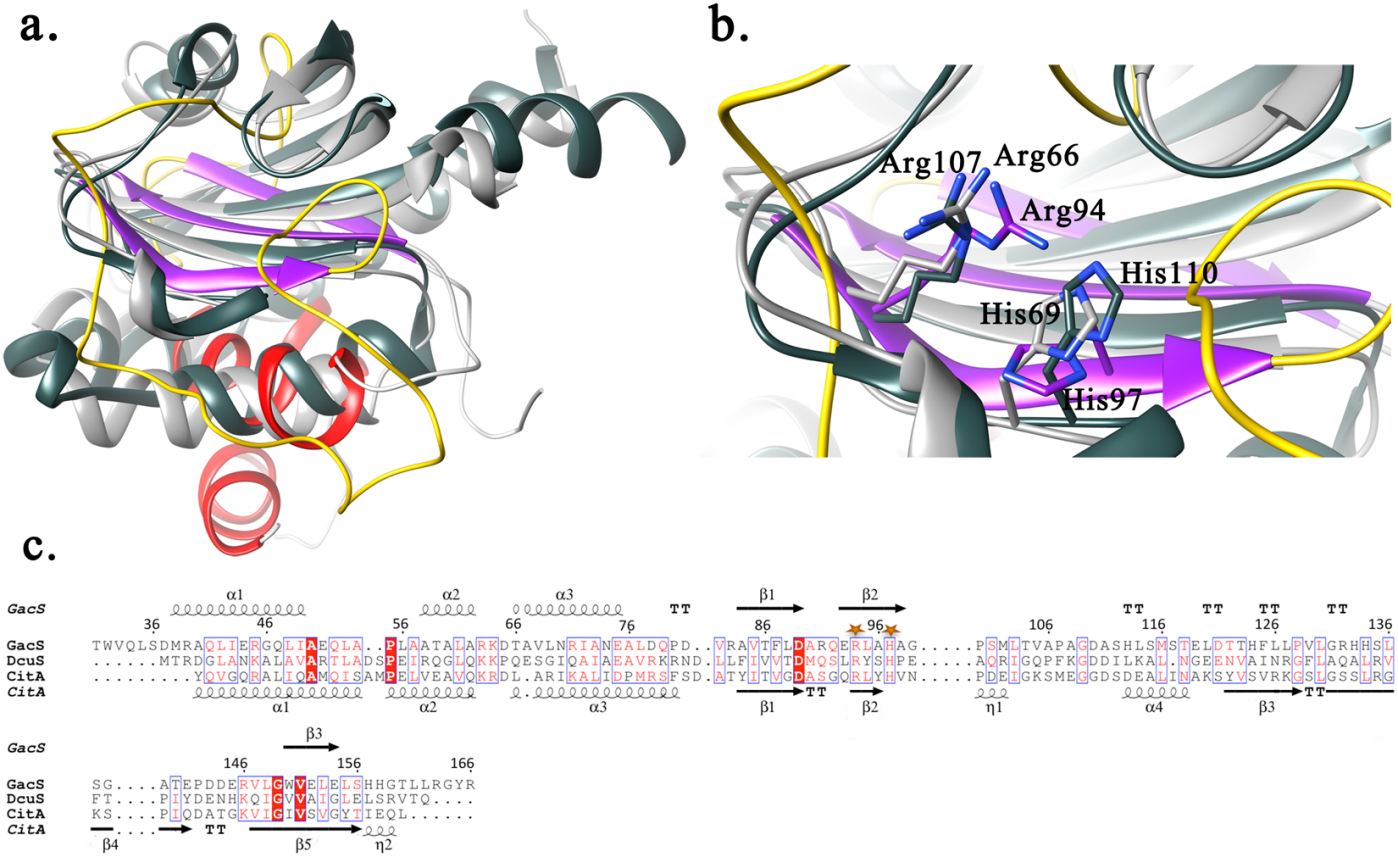
Structural comparison. (**a**) Superimposition of GacS_PD_ (colored secondary structure) and two structural homologues, *E. coli* DcuSp (dark grey), and *K. pneumonia* CitAp (light grey). (**b**) Close-up views of the conservation pattern made by the two positively-charged residues between GacS_PD_, CitAp and DcuSp. (**c**) Structural sequence alignment of GacS_PD_, CitAp and DcuSp. The conserved Arg and His residues are highlighted with orange stars.

A hidden Markov model (HMM)-based profile search identified several periplasmic domains adopting a PDC fold such as the CitA detector kinase (CitAp domain), the methyl-accepting chemotaxis protein from *Geobacter sulfurreducens* and the DcuS sensor kinase (DcuSp domain), as the closest structural homologs (HHpred true-positive Prob >97.4% Figs 5 and S4). Next, pairwise structural comparison between GacS_PD_ and CitAp, DcuSp, the *Salmonella typhimurium* metal binding domain (PhoQ), the *Halorhodospira halophile* photoactive yellow protein *(PYP)* and the *Geobacter sulfurreducens* methyl-accepting binding domains (GSU0582 and GSU0935) showed an average rmsd value of 2.78 ± 0.4 Å for 42 CAs atoms of the central β-sheet, compared to a value of 2.1 ± 0.5 Å between various PAS and PDC domains^34–42^.

Besides this overall fold conservation, the striking structural differences between GacS_PD_ and other PDC/PAS detector domains reside in the number of β-strands from the central β-sheet and the presence of a major loop (Gly99-Leu148) covering the outer face of the β-sheet at the apical side of GacS_PD_ (Fig. S4). In other PDC-type detector domains, such as CitAp, DcuSp and PhoQ, the corresponding loop shows high variability in length and is often limited to two small α-helices containing up to twelve residues that contribute to ligand specificity^43^ (Fig. S4). In fact, an overlay of PDC-containing domain structures reveals that GacS_PD_ has the longest, 49-residues length, loop region (Fig. 5a), which exhibits a pronounced flexibility in solution as demonstrated by NMR relaxation data. Such loop flexibility has been reported for other PDC homologues, such as the citrate-free form of CitAp, where a major disordered loop of more than 30 residues, not detected in the crystal structure, showed strong line broadening and low ^1^H/^15^N signals from backbone amide groups by NMR spectroscopy. Interestingly, stabilization of this loop occurs in the presence of citrate, that also promotes folding of the nearby C-terminal helix^44^. Thus, one could propose that conformational changes of the major loop could also occur in GacS_PD_ upon ligand binding, as evidenced for CitAp^44^.

**Figure 6:**
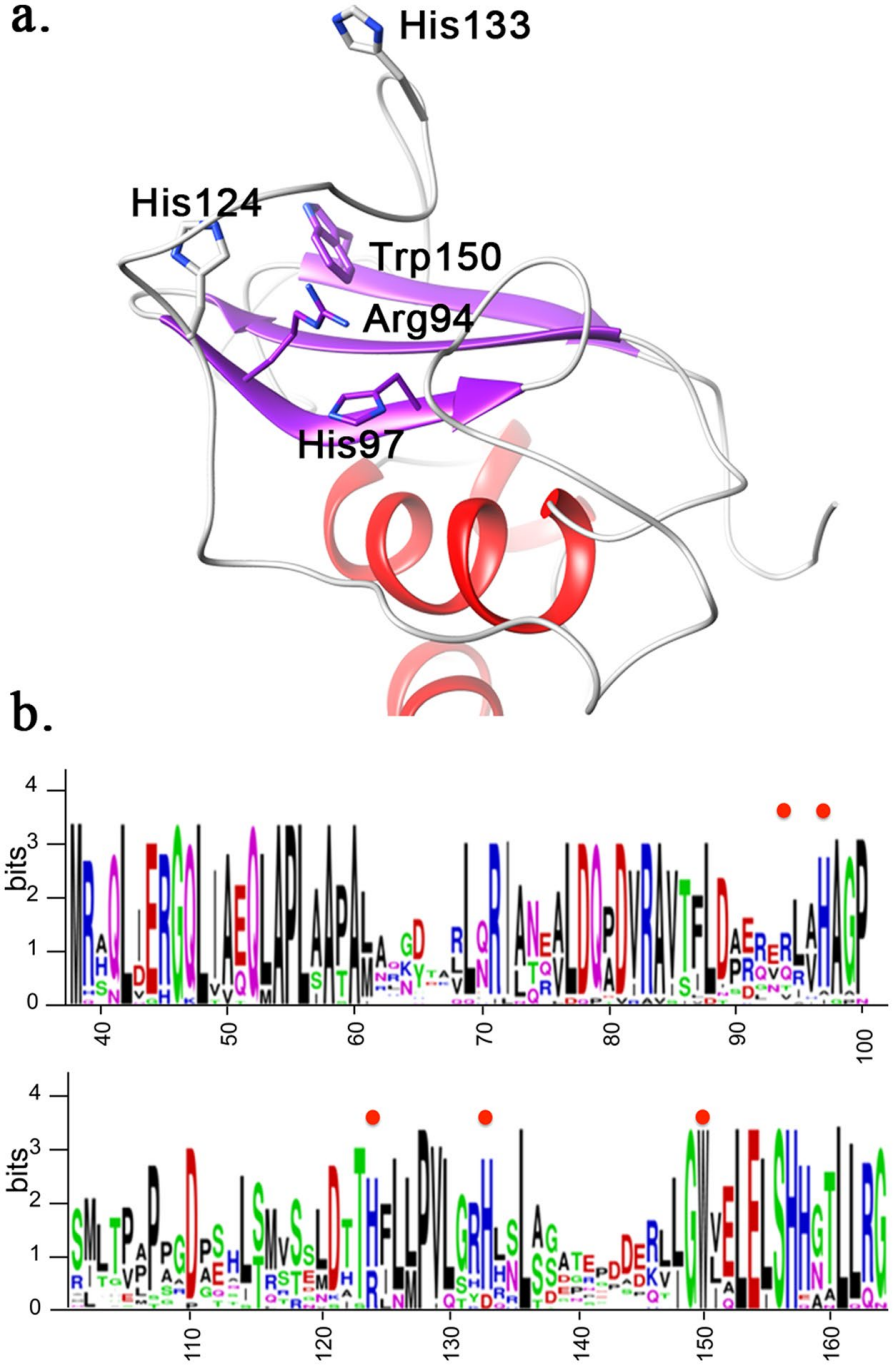
Mutation locations in GacS_PD_ solution structure. (**a**) A GacS_PD_ cartoon representation with the selected mutated residues shown as sticks. (**b**) Multiple sequence alignment of 227 GacS periplasmic detector domains from the *Pseudomonas* Genus. The logo representation was generated with the Skylign web-server^62^. Location of the mutated residues (Arg94, His97, His124, His133 and Trp150) is indicated by a red dot.

A sequence alignment of GacS_PD_ with the closest structural homologues CitAp and DcuSp evidences conservation of the GacS_PD_ Arg94 and His97 pair of interacting residues in these closest structural homologs. In fact, the corresponding residue pairs in DcuSp (Arg107, His110) and CitAp (Arg66, His69) are key residues for binding the negatively-charged C4 and C6-dicarboxyalte ligands, respectively (Fig. 5b and c)^35, 39^. Moreover, a multiple sequence alignment using 227 GacS periplasmic detector domains from the *Pseudomonas* genus reveals conservation of three residues (His97, His133 and Trp150) within the *Pseudomonas* genus, while Arg94 is not conserved. With the exception of the Trp150 indole ring stably anchored in the hydrophobic core, Arg94, His97 and His124 form a triad of interacting residues on top of the β-sheet facing the major loop and define a pocket that could represent a putative ligand-binding site (Figs 5 and 6). His124 is a conserved basic residue within the *Pseudomonas aeruginosa* group and can be substituted with an arginine (Fig. 6b). The solvent-exposed His133 is located within the major loop at one edge of this putative binding site. Given the striking conservation pattern of the Arg-His residue pair in GacS_PD_, we performed ^1^H-^15^N HSQC titration experiments of GacS_PD_ with a series of related negatively charged molecules, e.g. citrate, fumarate as well as other Krebs cycle intermediates reported to affect the GacS/GacA signaling pathway^45, 46^, and other molecules or ions (Table S5), but no binding with GacS_PD_ was observed.

### GacS_PD_ harbors a functional surface region within the major loop

To assess the functional role of these five GacS_PD_ residues, we first checked the impact of mutations into alanine on the GacS_PD_ fold using NMR ^1^H-^15^N HSQC experiments. With the exception of the GacS_PD_ W150A variant that was found to be misfolded due to its major role in GacS_PD_ folding and stability, the three fingerprints recorded for a GacS_PD_ double mutant (Arg94Ala and His97Ala) and each of the H124A and H133A single mutant did not reveal an alteration of the overall GacS_PD_ fold. compared to WT GacS_PD_ (Fig. S6).

**Figure 7:**
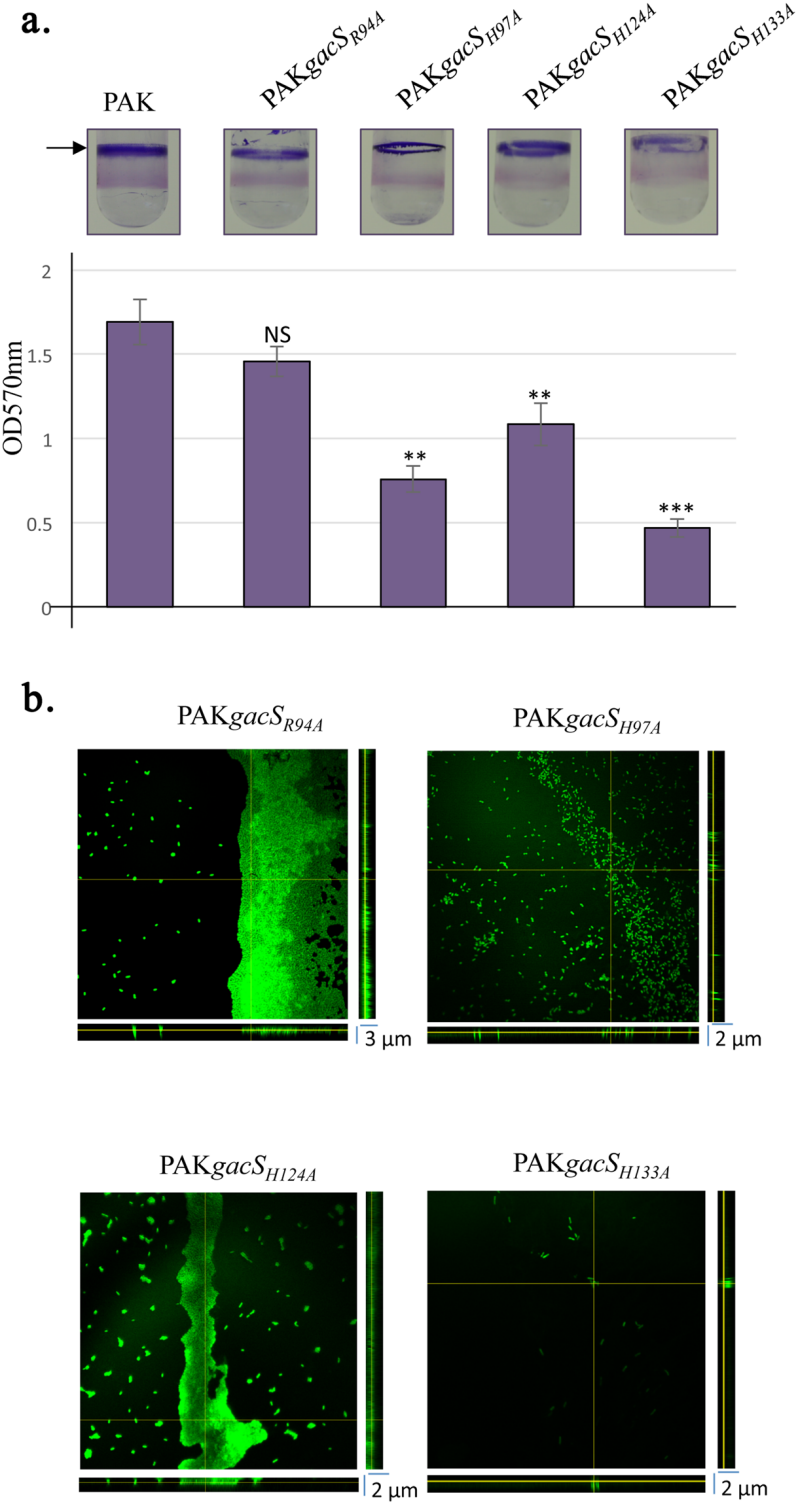
Effect of mutations in the GacS detection domain on biofilm formation. (**a**) Biofilm production in glass tubes (upper panel) is illustrated and quantified after crystal violet staining (lower panel). The corresponding levels of biofilm production represent means values (with error bars) obtained from three independent experiments. *, **, *** and ns referred to p < 0.05, p < 0.01 and p < 0.001 and non-significant difference, respectively, according to the Wilcoxon-Mann-Whitney tests. (**b**) Biofilm formation monitored by confocal laser scanning microscopy after 12 h. Extracted z images and their respective xy and xz planes are shown.

We next generated the four *gacS*_*R94A*_, *gacS*_*H97A*_, *gacS*_*H124A*_ and *gacS*_*H133A*_ mutant PAK strains to assess the potential role of these residues for biofilm formation. Combined analyzes using crystal violet staining and confocal microscopy revealed that the two GacS His97Ala and His133Ala mutants abolish biofilm formation compared to the WT PAK strain (Fig. 7). The biofilm morphology of these two *gacS*_*H97A*_ and *gacS*_*H133A*_ PAK strains exhibits a strong reduction compared to the WT PAK strain (Figs 1b and 7b), corresponding to single isolated colonies as observed for the Δ*gacS* PAK strain (Fig. 1b). The *gacS*_*H124A*_ mutant PAK strain showed an altered biofilm morphology compared to the WT strain, indicating a functional role of the pocket-lining His124 residue in biofilm formation. By contrast, the *gacS*_*R94A*_ mutant PAK strain showed biofilm morphology similar to WT PAK strain (Figs 1 and 7b), indicating that mutating Arg94 does not impair biofilm formation.

**Figure 8:**
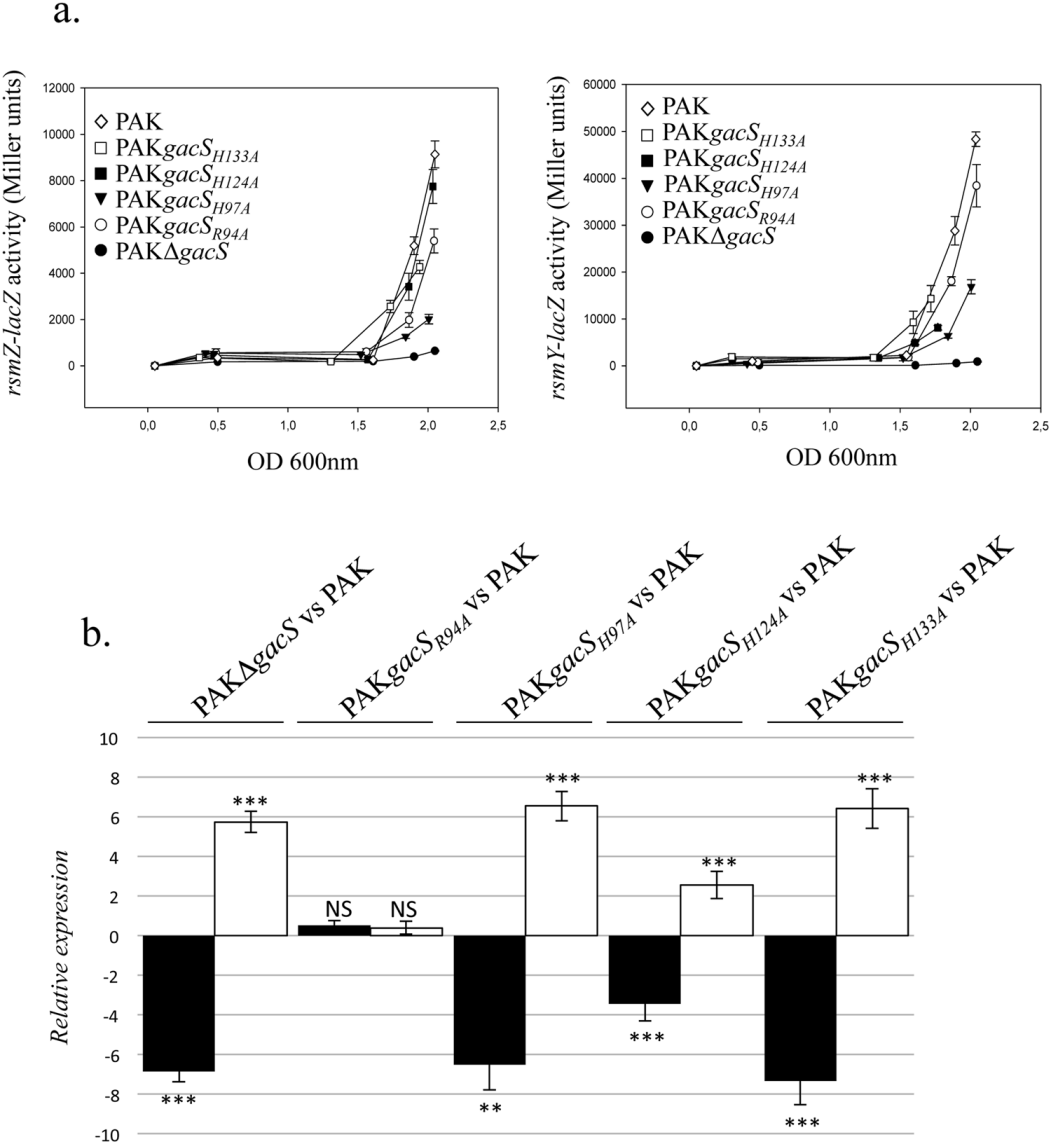
Effect of mutations in the GacS detection domain on *rsm* genes, T3SS and T6SS expression. (**a**) Activities of the rsmZ–lacZ (left panel) and rsmY–lacZ (right panel) transcriptional chromosomal fusions were monitored at different growth stages in the PAK_WT_, PAKΔ*gacS*, PAK*gacS*_*R94A*_, PAK*gacS*_*H97A*_, *PAKgacS*_*H124A*_ *PAKgacS*_*H133A*_ strains. The corresponding β-galactosidase activities are expressed in Miller units and correspond to mean values (with error bars) obtained from three independent experiments. (**b**) Transcript levels of VgrG1b (T6SS; black bar) and ExoS (T3SS; white bar) were monitored in the PAK, PAKΔ*gacS*, PAK*gacS*_*R94A*_, PAK*gacS*_*H97A*_, *PAKgacS*_*H124A*_ and *PAKgacS*_*H133A*_ strains using RT-qPCR and fold change is displayed for the four mutant strains compared to the PAK strain. *, ** and *** refer to p < 0.05, p < 0.01 and p < 0.001, respectively, according to the moderated t-tests.

To further confirm the functional role of these residues, we examined the effect of these four GacS_PD_ mutants on the expression of the GacS/GacA TCS target genes, as already performed with the GacSΔ_PD_ PAK strain for monitoring *rsmZ*/*rsmY* expression and T6SS and T3SS activation or repression. In turn, the *rsmY–lacZ* and *rsmZ–lacZ* transcriptional fusions were introduced into the four *gacS*_*R94A*_, *gacS*_*H97A*_, *gacS*_*H124A*_ and *gacS*_*H133A*_ mutant PAK strains. Expression level of the two RsmY and RsmZ sRNAs, as measured by the β-galactosidase activity assay at various growth stages, was decreased in the three *gacS*_*H97A*_, *gacS*_*H124A*_ and *gacS*_*H133A*_ mutant PAK strains by around 4.6-fold, 1.7-fold and 2.2-fold, respectively for RsmZ and around 3-fold, 5.7-fold and 6-fold, respectively for RsmY, compared to the WT PAK strain (Fig. 8a). In parallel we also examined whether the *gacS*_*R94A*_, *gacS*_*H97A*_, *gacS*_*H124A*_ and *gacS*_*H133A*_ mutant strains could affect expression of T3SS and T6SS. By monitoring the expression level of T6SS (vgrG1b) or T3SS (exoS), we found a 6.2, 2.3 and 6.1-fold induction of T3SS associated to a 6.3, 3.8 and 7.1-fold repression of T6SS in the *gacS*_*H97A*_, *gacS*_*H124A*_ and *gacS*_*H133A*_ mutants, respectively (Fig. 8b).

In summary, we unveiled a functional surface region within GacS_PD_ involved in the activation of the GacS/GacA signaling pathway in *P aeruginosa*, arguing for a functional role of the periplasmic detector domain of the central GacS HK. The GacS_PD_ NMR solution structure reveals an atypical PDC/PAS-like domain fold that consists of a 3-stranded β-sheet flanked by 3 α-helices and a major loop. This functional region consists of a positively-charged pocket at the apical side and is defined by at least the three residues His97 H124, and H133 residues, which clearly involves the major loop and one face of the central β-sheet. Interestingly, while mutation of the invariant Arg94 residue in CitA/DcuS/GacS did not affect the *P. aeruginosa* adherence, mutation of the three His97, H124 and His133 GacS residues, the latter two being not conserved in CitA/DcuS, severely altered biofilm formation. We could thus propose that the ligand binding site in GacS_PD_ does not occur at the same site as the closest structural homologues CitAp and DcuSp. This is consistent with a functional diversity of the GacS periplasmic detector domain acquired during evolution of the PDC/PAS domain-containing HK.

## Materials Methods

### Bacterial strains and culture conditions

Strains and plasmids used for this study are listed in Tables S1 and S3, respectively. Strains were grown at 37 °C in LB medium or in M63 medium supplemented with 1 mM MgCl_2_, 0.5% casa-aminoacids, 0.2% glucose. Recombinant plasmids were introduced into *P. aeruginosa* genome through conjugative transfer using pRK2013. Transconjugants were selected on *Pseudomonas* isolation agar (PIA;Difco Laboratories) supplemented with appropriate antibiotics. Kanamycin (Km) at 50 µg/ml and tetracycline (Tc) at 150 µg/ml supplemented with streptomycin^47^ at 2 mg/ml were used for *E. coli* and *P. aeruginosa* strains, respectively.

### Construction of chromosomal variants and mutants

To generate a gacSΔ_PD_ PAK strain harbouring a chromosomal copy of a *gacS* variant devoid of a periplasmic domain, DNA fragments corresponding to the upstream and downstream sequences (approximately 500 pb) of the deleted region were amplified from the PAK genomic DNA using appropriate oligonucleotide pairs (Table S4). The PCR products were cloned into the pCR2.1 vector (Invitrogen) using the SLIC method. After DNA sequencing, the cloned region was digested by BamH1 and ApaI and inserted into the linearized pKNG101 vector, resulting in the PKNG101 *gacS*Δ_*PD*_ plasmid.

To engineer the four *gacS*_*R94A*_, *gacS*_*H97A*_, *gacS*_*H124A*_, *gacS*_*H133A*_, mutant PAK strains, each harbouring a point alanine mutation into a chromosomal copy of the *gacS* gene, the upstream and downstream sequences (approximately 500 pb) were amplified from the PAK genomic DNA using the appropriate pairs of primers (Table S4). The forward and reverse PCR products were linked and cloned into the suicide pKNG101 vector using the SLIC strategy, yielding the four *gacS*_*R94A*,_ *gacS*_*H97A*_, *gacS*_*H124A*_ and *gacS*_*H133A*_ PKNG101 plasmids. The resulting suicide plasmid was introduced into the *P. aeruginosa* genomic DNA through conjugative transfer by a three-partner procedure using pRK2013. The deletion mutants were obtained by a double selection: first on LB agar supplemented with Irgasan (25 μg/mL) and streptomycin (1000 μg/mL) at 37 °C followed by a NaCl-free LB agar containing 6% sucrose at 30 °C. The genome of each mutant was extracted and the targeted DNA fragment was amplified and verified by sequencing.

### RT-qPCR

The WT, Δ*gacS*, *gacS*Δ_PD_, *gacS*_*R94A*_, *gacS*_*H97A*_, *gacS*_*H124A*_, *gacS*_*H133A*_ PAK strains were grown at 37 °C under agitation until OD_600_ reached 4. Total cellular RNA from a 10 mL culture medium was isolated using the pureYield RNA midiprep system (Promega), cleaned up and concentrated using the RNeasy kit (Qiagen). RNA yield, purity and integrity were further evaluated on Nanodrop and Experion devices. Reverse transcription was performed on 2 μg of RNA using the SuperScript III first-strand synthesis system (Invitrogen). Real-time PCR runs were carried out on a CFX96 Real-Time System (Bio-Rad). Cycling parameters of the real-time PCR were 98 °C for 2 min, followed by 45 cycles of 98 °C for 5 s and 60 °C for 10 s, ending with a melting curve from 65 °C to 95 °C to evaluate amplification specificity. To determine the amplification kinetics of each product, fluorescence derived from incorporation of EvaGreen into the double-stranded PCR products was measured at the end of each cycle using a SsoFast EvaGreen Supermix 2X Kit (Bio-Rad). The data were analyzed using Bio-Rad CFX Manager Software 3.0 (Bio-Rad). The *uvrD* gene was used as a reference for normalization, in particular because transcription of *uvrD* is fairly stable in bacteria exposed to antibiotics even at relatively high concentrations^48^.

### Biofilm assay

The *P*. *aeruginosa* adherence assay was performed in individual glass tubes containing 1 mL of medium as described previously^31^. Bacteria were grown in M63 medium supplemented with 1 mM MgCl_2_, 0.5% casa-amino acids, 0.2% glucose under static conditions at 30 °C. After 12 hours, the cultures were incubated with 1% Crystal Violet for 10 min to stain the attached bacteria and washed twice. Staining was extracted by treatment with 400 μL 95% ethanol. Subsequently, 600 μL of water were added and OD_570_ was measured. All quantification assays were performed at least in triplicate.

### Measurements of β-galactosidase activity

Strains carrying the *lacZ* transcriptional fusions were grown at 37 °C in agitated LB medium. Bacterial cells were collected by centrifugation at different growth times. The β-galactosidase activity was measured using the method of Miller^49^.

### Confocal laser scanning microscopy analysis of biofilm

*P*. *aeruginosa* strains were grown in an 8-well chambered coverglass (Lab-teK II). 200 μL of M63 derivate medium containing a bacterial suspension at an OD_600_ of 0.1 was incubated at 30 °C. After 12 h, the medium was aspired from the corner of each chamber and rinsed twice by adding 200 μL of sterile PBS to remove unattached cells. Prior to observation, bacteria were fixed with 4% paraformaldehyde and stained using 4′,6′-diamidino-2-phenylindole (DAPI) for 15 min. Images were taken at locations of biofilm formation using a confocal laser scanning microscope Confocal Olympus FV1000. Positions were chosen for sagittal sections (xz position) to minimize experimental variability. The number of images for the 3D biofilm observation within each stack depended on biofilm thickness. All confocal images were analysed using the imageJ software^50^.

### Cloning, expression and purification of GacS_PD_ for structural determination

The DNA sequence coding for the periplasmic detector domain of the GacS HK (Met38-Gly164) was amplified from the extracted PAK genome by using the appropriate primers (Table S4). The PCR products were cloned into the pLic03 vector linearized by BamH1 using the LIC method^51^ yielding the pLic03_GacSsp coding for GacS_PD_ fused to a 6xHis tag and a tobacco etch virus (TEV) at the N terminal region. Expression and purification of GacS_PD_ were performed as described^33^.

### Structure calculation

The NMR sample contained 0.8 mM protein concentration (90% H2O, 10% D2O) in 150 mM NaCl, 50 mM phosphate buffer, pH7. Recorded spectra were analyzed with CARA on the basis of the previously published backbone amide and side chain resonances assignment^33^. The approximate inter-proton distances were obtained from the 2D NOESY, ^13^C NOESY-HSQC and 3D ^15^N NOESY-HSQC spectra recorded using a mixing time of 150 ms. A total number of 1299 restraints were used for structure calculations. Hydrogen bonds were identified by recording long-range J_NC’_ HNCO-COSY^52^ and a series of ^15^N-^1^H HSQC spectra using a sample freshly dissolved in D_2_O; 27 nonexchangeable amides were located in regions of defined secondary structures. Hydrogen bond constraints were introduced using distance restraints in the range of 2.7–3.0 Å and 1.8–2.0 Å for O-N and O-HN, respectively. Moreover, 94 additional dihedral restraints were calculated using TALOS+ based on ^1^H^α^, ^13^C^α^, ^13^C^β^, ^13^C’ and ^15^N chemical shifts^53^. Input data and structure calculation statistics are summarized in Table S2. The accuracy of the NMR models has been assessed based on the traditional criteria for successful structure calculation using the program CYANA^54^. 100 structures were generated and the 20 lowest-energy structures were selected and each subjected to restrained molecular dynamics using the Amber 4.1 force field within the SANDER module of Amber 10. Water molecules were stripped off and energy terms were calculated for the protein using AMBER^55^. Non-bonded interaction cutoff was 15 Å for the restrained MD runs. The final structural ensemble was analysed using PROCHECK-NMR^56^. Structure coordinates have been deposited to the Protein Data Bank under accession number 5O7J.

### Relaxation study

^15^N *T*_1_, *T*_2_, and NOE NMR relaxation measurements were performed at 25 °C on a Bruker Avance 600 MHz using a 2 mM GacS_PD_ sample (90% H2O, 10% D2O) in 150 mM NaCl, 50 mM phosphate buffer, pH 7.0. *T*_1_ data and *T*_2_ data were both acquired with ten relaxation delays (10, 20, 50, 100, 200, 300, 400, 600, 800 and 1000 ms and 17.6, 35.2, 52.8, 70.4, 88.0, 105.6, 123.2, 140.8, 158.4 and 176.0 ms, respectively). Experimental ^15^N heteronucleaur NOE values were determined from the intensity ratios of amide signals of interleaved 2D ^1^H-^15^N HSQC spectra with and without a 5 sec saturation period. Relaxation times were calculated as reported by Farrow and coworkers^57^ using an exponential fit of single exponential decays to peak intensity values: *I* = *I*_0_exp(−t/T) where *T* = *T*_1_ or *T*_2_, and *I* = resonance intensity at time *t*. Steady-state ^1^H−^15^N NOE ratios were calculated using the *r* = *I/I*_0_ expression.

### Ligand binding using ^1^H-^15^N HSQC titration

Putative ligand molecules (see Table S5) were tested at 298 K using 150 μM of ^15^N-labelled GacS_PD_ native protein and mutants (50 mM Na_2_HPO_4_/NaH_2_PO_4_ buffer pH 7, 50 mM NaCl) with a ligand concentration range of 0.1–20 mM.

### Bioinformatics analysis

227 GacS protein sequence homologues belonging to the Pseudomonas genus were retrieved from Uniprot (ftp://ftp.uniprot.org, Feb 2017) and aligned with Muscle^58^ and downloaded from the Uniprot FTP server (ftp://ftp.uniprot.org) in February, 2017. From the multiple alignments, a selection of GacS periplasmic domains was made with Gblock Server^59^. Maximum likelihood trees were generated with PhyML-SMS^60^ using the LG empirical amino acid substitution model of evolution^61^ and 1000 bootstrap replicates. In parallel, the resulting trees were compared with that obtained from the downloaded 16S rRNA sequences. The tree parameters including topology were optimized. The sequences of the periplasmic domains that belong to the same branch as *P. aeruginosa* GacS were retained to generate the sequence logo using the Skylign web-server^62^.

## Electronic supplementary material

Supplementary infos
