# Supplementary material for: Structural and functional insights into the periplasmic detector domain of the GacS histidine kinase controlling biofilm formation in Pseudomonas aeruginosa
Source: Sci Rep. 2017 Sep 12;7:11262. doi: 10.1038/s41598-017-11361-3 (PMC5595915; doi:10.1038/s41598-017-11361-3)
Supplement: Supplementary file 1 — Supplementary infos [file 41598_2017_11361_MOESM1_ESM.pdf]

Structural and functional insights into the periplasmic detector domain of the GacS histidine kinase controlling biofilm formation in *Pseudomonas aeruginosa*

Ahmad Ali-Ahmad[1*], Firas Fadel, Corinne Sebban-Kreuzer, Moly Ba, Gauthier Dangla Pélissier, Olivier Bornet, Françoise Guerlesquin, Yves Bourne, Christophe Bordi, Florence Vincent

Table S1: Strains used in this study

Strains	Relevant characteristics	Source
E. coli		
DH5α	*endA1 hsdR17 supE44 thi-1 recA1 gyrA relA1 Δ(lacZYA-argF)U169 deoR (phi 80lacZ Δ M15)*	Lab collection
CC118(λpir)	Host strain for pKNG101 replication, Δ(*ara-leu*) *araD* Δl*ac*X74 *galE galK phoA20 thi-1 rpsE rpoB argE*(Am) *recA1* Rf^R (λpir)	Lab collection
BL21 DE3	*fhuA2 [lon] ompT gal (λ DE3) [dcm]* Δ*hsdS* *λ DE3 = λ sBamHIo* Δ*EcoRI-B int::(lacI::PlacUV5::T7 gene1) i21* Δ*nin5*	Lab collection
P. aeruginosa		
PAK	Wild type	[63]
PAKΔ*gacS*	PAK deletion mutant for *gacS* gene	[31]
PAK*gacS*Δ$_{PD}$	PAK deletion mutant for the periplasmic domain of *gacS* gene	This study
PAK*gacS*$_{R94A}$	Point chromosomal mutant R94A of *gacS* gene in PAK	This study
PAK*gacS*$_{H97A}$	Point chromosomal mutant H97A of *gacS* gene in PAK	This study
PAK*gacS*$_{D110A}$	Point chromosomal mutant D110A of *gacS* gene in PAK	This study
PAK*gacS*$_{H124A}$	Point chromosomal mutant H124A of *gacS* gene in PAK	This study
PAK*gacS*$_{H133A}$	Point chromosomal mutant H133A of *gacS* gene in PAK	This study
PAK*gacS*$_{W150A}$	Point chromosomal mutant W150A of *gacS* gene in PAK	This study
PAKΔRetS	PAK deletion mutant for *retS* gene	[14]
PAK*attB::rsmY-lacZ*	PAK strain with *rsmY-lacZ* inserted at *attB* sites	[17]
PAK*attB::rsmZ-lacZ*	PAK strain with *rsmZ-lacZ* inserted at *attB* sites	[17]
PAKΔ*gacSattB::rsmY-lacZ*	PAKΔ*gacS* strain with *rsmY-lacZ* inserted at *attB* sites	[17]
PAKΔ*gacSattB::rsmZ-lacZ*	PAKΔ*gacS* strain with *rsmZ-lacZ* inserted at *attB* sites	[17]
PAKΔ*gacS*$_{PD}$*attB::rsmY-lacZ*	PAKΔ*gacS*$_{PD}$ strain with *rsmY-lacZ* inserted at *attB* sites	This study
PAKΔ*gacS*$_{PD}$*attB::rsmZ-lacZ*	PAKΔ*gacS*$_{PD}$ strain with *rsmZ-lacZ* inserted at *attB* sites	This study
PAK*gacS*$_{R94A}$*attB::rsmY-lacZ*	PAK*gacS*$_{R94A}$ strain with *rsmY-lacZ* inserted at *attB* sites	This study
PAK*gacS*$_{R94A}$*attB::rsmZ-lacZ*	PAK*gacS*$_{R94A}$ strain with *rsmZ-lacZ* inserted at *attB* sites	This study
PAK*gacS*$_{H97A}$*attB::rsmY-lacZ*	PAK*gacS*$_{H97A}$ strain with *rsmY-lacZ* inserted at *attB* sites	This study
PAK*gacS*$_{H97A}$*attB::rsmZ-lacZ*	PAK*gacS*$_{H97A}$ strain with *rsmZ-lacZ* inserted at *attB* sites	This study
PAK*gacS*$_{D110A}$*attB::rsmY-lacZ*	PAK*gacS*$_{D110A}$ strain with *rsmY-lacZ* inserted at *attB* sites	This study
PAK*gacS*$_{D110A}$*attB::rsmZ-lacZ*	PAK*gacS*$_{D110A}$ strain with *rsmZ-lacZ* inserted at *attB* sites	This study
PAK*gacS*$_{H124A}$*attB::rsmY-lacZ*	PAK*gacS*$_{H124A}$ strain with *rsmY-lacZ* inserted at *attB* sites	This study
PAK*gacS*$_{H124A}$*attB::rsmZ-lacZ*	PAK*gacS*$_{H124A}$ strain with *rsmZ-lacZ* inserted at *attB* sites	This study
PAK*gacS*$_{H133A}$*attB::rsmY-lacZ*	PAK*gacS*$_{H133A}$ strain with *rsmY-lacZ* inserted at *attB* sites	This study
PAK*gacS*$_{H133A}$*attB::rsmZ-lacZ*	PAK*gacS*$_{H133A}$ strain with *rsmZ-lacZ* inserted at *attB* sites	This study
PAK*gacS*$_{W150A}$*attB::rsmY-lacZ*	PAK*gacS*$_{W150A}$ strain with *rsmY-lacZ* inserted at *attB* sites	This study

Table S2: NMR and refinement statistics of the GacSp structure. Structural statistics and restraint violations of the 20 selected structures representative of GacSp in solution at 298K**.**

**Restraints used for the calculation**	
NMR distance and dihedral restraints	
Effective distance restraints	1299
Short-range ≤(|i-j|1)	613
Medium-range <<(1|i-j|5)	278
Long-range, |i-j|≥5	408
Average number of restraints per residue	9.48
Dihedrals	94
Hydrogen bonds	27
Structure statistics	
Distance restraint violation>0.5Å	0
CNS potential energy (kcal/mol)	
E_{total}	-4077.645
E_{bond}	89.358
E_{angle}	485.937
$E_{dihedral}$	1398.193
E_{VdW}	-1046.495
$E_{electric}$	-8029.448
Average ensemble RMSD Å	
Backbone (residues 38-164)	0.95
Heavy atoms (residues 38-164)	1.44
Ramachandran plot %*	
Most favorable region	68.1
Additiona allowed region	27.4
Generous allowed region	3.5
Disallowed region	0.9
PDB ID: 5O7J	

* Calculated using PROCHECK program implanted in PDBsum server

Table S3: Plasmids used in this study

Plasmids	Relevant characteristics*	Source
pLic03	Vector containing T7 promoter with pBR322 origin of replication, Km^R	[64]
pLic03_GacSsp	pLic03 carrying the Nt-6his tagged GacSsp subdomain DNA region	This study
PCR2.1	TA cloning vector for PCR products, LacZα ColE1 f1 ori, Ap^R, Kam^R	Invitrogen
PKNG101	Suicide vector in P. aeruginosa; SacB, Sm^R	[65]
pKRK2013	Tra^+ Mob^+ Km^+	Lab collection
pKNG101Δ$gacS_{PD}$	Mutator plasmid for GacS periplasmic domain deletion in gacS gene, Sm^R	This study
pKNG101$gacS_{R94A}$	Mutator plasmid for point mutation R94A in gacS gene, Sm^R	This study
pKNG101$gacS_{H97A}$	Mutator plasmid for point mutation R97A in gacS gene, Sm^R	This study
pKNG101$gacS_{D110A}$	Mutator plasmid for point mutation D110A in gacS gene, Sm^R	This study
pKNG101$gacS_{H124A}$	Mutator plasmid for point mutation H124A in gacS gene, Sm^R	This study
pKNG101$gacS_{H133A}$	Mutator plasmid for point mutation H133A in gacS gene, Sm^R	This study
pKNG101$gacS_{W150A}$	Mutator plasmid for point mutation W150A in gacS gene, Sm^R	This study
miniCTX-lacZ	TC lacZ+ ; self-proficient integration vector with tet, V-FRT-attPMCS, ori, int, and oriT	[66]
miniCTX-*rsmY-lacZ*	Promoter region of *rsmY* gene inserted into miniCTX-*lacZ*, Tc^R	[17]
miniCTX-*rsmZ-lacZ*	Promoter region of *rsmZ* gene inserted into miniCTX-*lacZ*, Tc^R	[17]

*Ap^R ampicillin resistance, Sm^R streptomycin resistance, TC^R tetracyclin resistance, Km^R Kanamycin

Table S4: Oligonucleotides used in this study

Names	Oligonucleotides (5'→3')
Proteins production	
UpGacS$_{PD}$	TACTTCCAATCAATGATGCGCGCCCAGTTGATCGAG
DnGacS$_{PD}$	TATCCACCTTTACTGTTATTATATCCGCGCAGCAGAGTCCCG
Point chromosomal mutations	
UpUGacS$_{R94A}$	CTACTTATGGTACCCGGGCGTCGCAAGCCGAATCCGCC
UpDGacS$_{R94A}$	CATGGGCGAGGGCTTCCTGGCGG
DnUGacS$_{R94A}$	CCGCCAGGAAGCCCTCGCCCATG
DnDGacS$_{R94A}$	CCTGCAGGTCGACGGATCGCCAGAGGCCAGTTCGTCCA
UpUGacS$_{H97A}$	CTACTTATGGTACCCGGGCGTCGCAAGCCGAATCCGCC
UpDGacS$_{H97A}$	GCTTGGCCCGGCGCGGGCGAGGCGTTCC
DnUGacS$_{H97A}$	GGAACGCCTCGCCCGCGCCGGGCCAAGC
DnDGacS$_{H97A}$	CCTGCAGGTCGACGGATCGCCAGAGGCCAGTTCGTCCA
UpUGacS$_{D110A}$	CTACTTATGGTACCCGGGGATTGATTAGCAATCGTG
UpDGacS$_{D110A}$	CATGCTCAAATGGCTGGCCGCGCCGGCCGGGGCGACGG
DnUGacS$_{D110A}$	CCGTCGCCCCGGCCGGCGCGGCCAGCCATTTGAGCATG
DnDGacS$_{D110A}$	CCTGCAGGTCGACGGATCGCCTCCTTGCGCGCCAGG
UpUGacS$_{H124A}$	CTACTTATGGTACCCGGGCGTCGCAAGCCGAATCCGCC
UpDGacS$_{H124A}$	AAGAACCGGTAGCAGGAACGCCGTGGTGTCCAGTTCGGTG
DnUGacS$_{H124A}$	CACCGAACTGGACACCACGGCGTTCCTGCTACCGGTTCTT
DnDGacS$_{H124A}$	CCTGCAGGTCGACGGATCATGTTGGCGAGGAACTCGGACT
UpUGacS$_{H133A}$	CTACTTATGGTACCCGGGCGTCGCAAGCCGAATCCGCC
UpDGacS$_{H133A}$	GTGGCGCCGGACAGGCTGTGCGCGCGGCCAAGAACCGGTA
DnUGacS$_{H133A}$	TACCGGTTCTTGGCCGCGCGCACAGCCTGTCCGGCGCCAC
DnDGacS$_{H133A}$	CCTGCAGGTCGACGGATCGCAGGTTGGTGAAACCGAGGAT
UpUGacS$_{W150A}$	CTACTTATGGTACCCGGGAACGTATGGCCGGGGAGC
UpDGacS$_{W150A}$	CGACAGTTCCAGTTCGACTGCGCCGAGTACGCGCTCGTC
DnUGacS$_{W150A}$	GACGAGCGCGTACTCGGCGCAGTCGAACTGGAACTGTCG
DnDGacS$_{W150A}$	CCTGCAGGTCGACGGATCCGCGGGCTGAGCTCGCTC
Deletion mutant	
UpUΔ*gacS*$_{PD}$	CGGGATCCCTTCGGCAGATAATCGTCGG
UpDΔ*gacS*$_{PD}$	CGTGGTGCGACCCGCGCTCGATCAACTGGG
DnUΔ*gacS*$_{PD}$	CGAGCGCGGGTCGCACCACGGGACTCTGCTGC
DnDΔ*gacS*$_{PD}$	CGGGGCCCATTTCTGGATGGTCGTGAGG
RT-qPCR	
uvrDUp	CACGCCTCGCCCTACAGCA
uvrDDn	GGATCTGGAAGTTCTCGCTCAGC
exoSUp	CTCTACACCGGCATTCACTA
exoSDn	CTTCACTACCTGTTCAGCCT
vgrG1bUp	CCATTTCTACGACTGGCACA
vgrG1bDo	GGGTAGTCGTACAAGCG

Figure S1

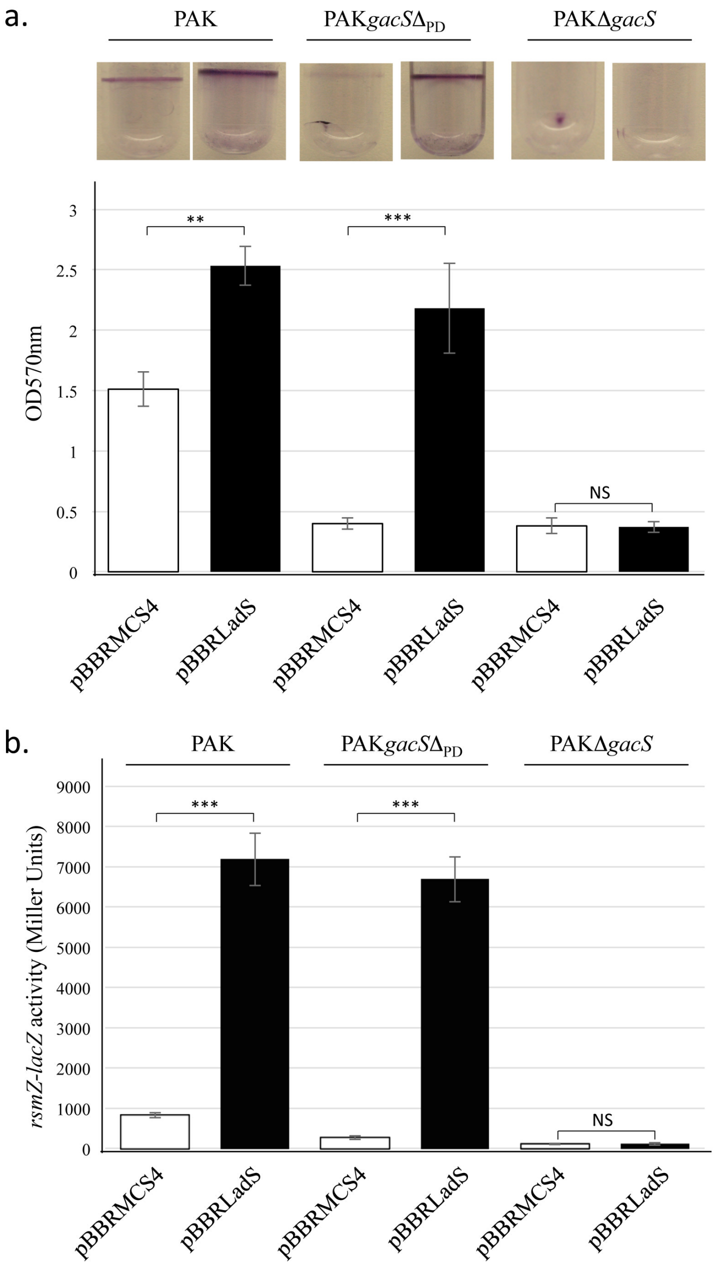

Figure S1: Effect of overexpression of full length LadS on biofilm formation in PAK, PAKΔ*gacS* and PAKΔ*gacS*Δ_{PD} strains. The pBBRladS plasmid containing the *ladS* HK gene (dark bar) and the corresponding pBBRMCS4 empty cloning vector (light bar) were conjugated in the PAK, PAKΔ*gacS*Δ_{PD} or PAKΔ*gacS* strains. **(a)** Biofilm production in glass tubes was illustrated (upper panel) and quantified after crystal violet-staining (lower panel). Levels of biofilm production represent mean values and standard deviations obtained from three independent experiments. **(b)** Activities of the *rsmZ*–lacZ transcriptional chromosomal fusions were monitored after 6 hours of growth (OD600nm ≈ 2) and corresponding β-galactosidase activities are expressed in Miller units and correspond to mean values (with error bars) obtained from three independent experiments. Wilcoxon-Mann-Whitney tests were performed and **, *** and ns referred to $p<0.01$, $p<0.001$ and non significant difference, respectively.

Figure S2

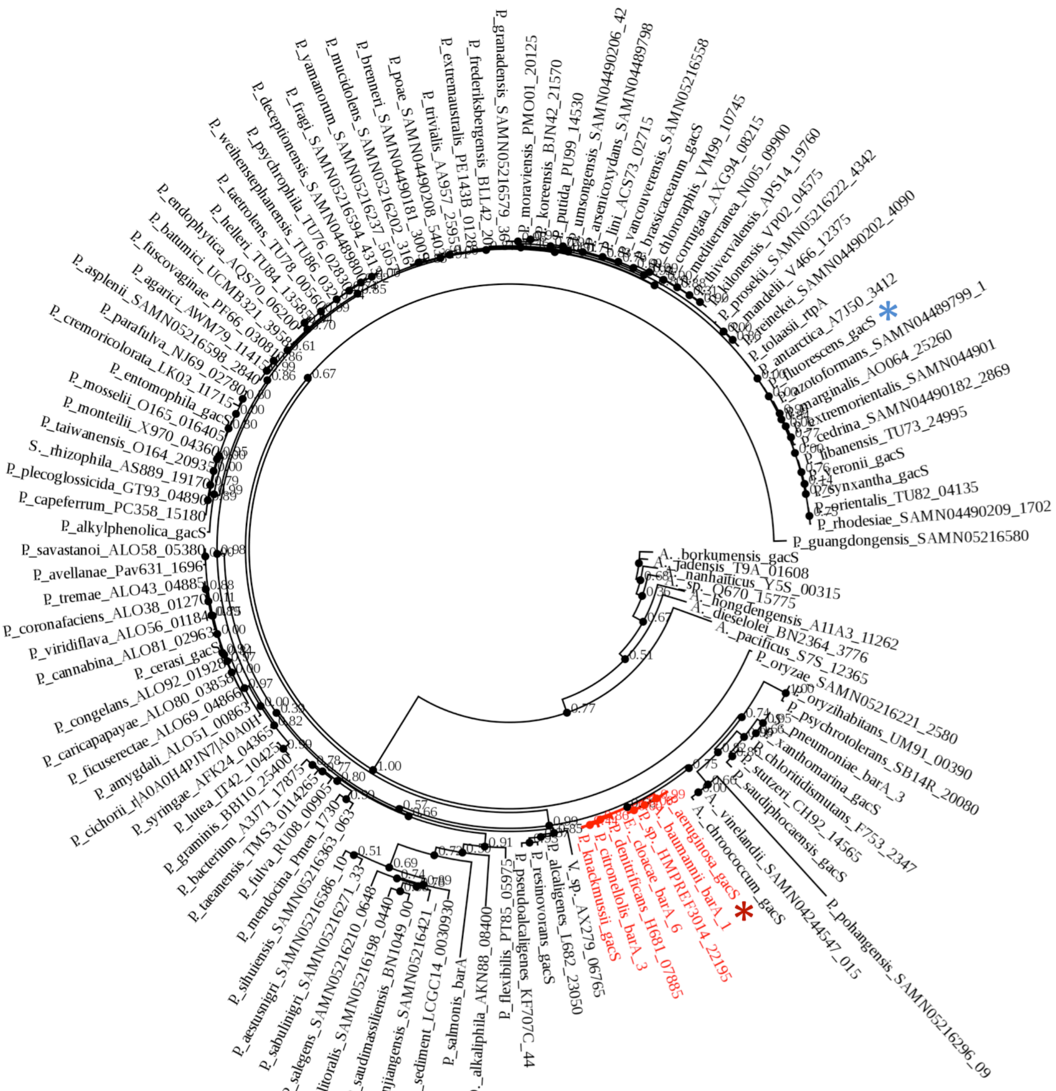

Figure S2: Phylogenetic tree of the Pseudomonas GacS periplasmic domain. The sequences are named according to the nomenclature adopted for *P. aeruginosa*. We calculated 1000 bootstrap replicates: bootstrap values > 0.5 (I.E 50 %) are indicated on branches. The main branch containing the *P. aeruginosa* GacS protein is highlighed in red, and the red and blue stars denote the position of the GacS periplasmic domain of *P. aeruginosa* and *P. fluorescens*, respectively.

Figure S3

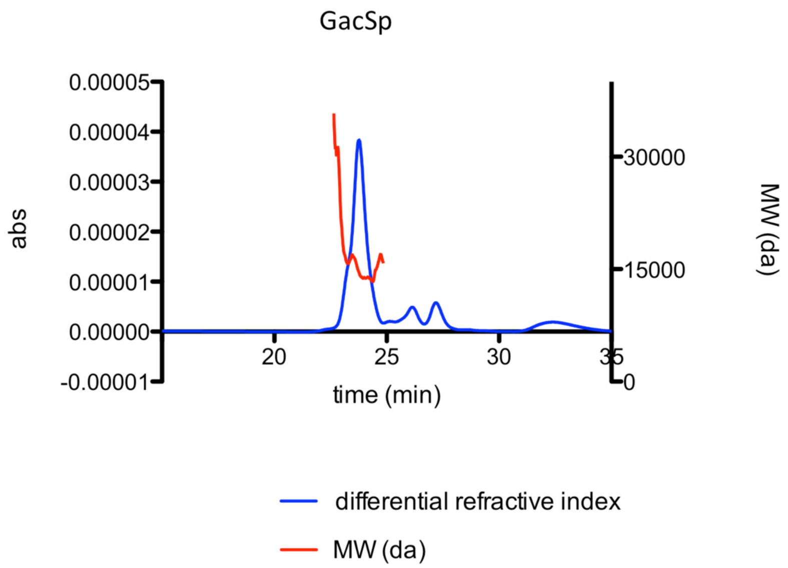

Figure S3: SEC-MALS elution profile of GacS$_{PD}$. The elution profile of GacS$_{PD}$ sample (blue line) and the estimated molecular weight (MW) (red line) reveals a dominant single population with an estimated molecular weight of 15000 Da.

Figure S4

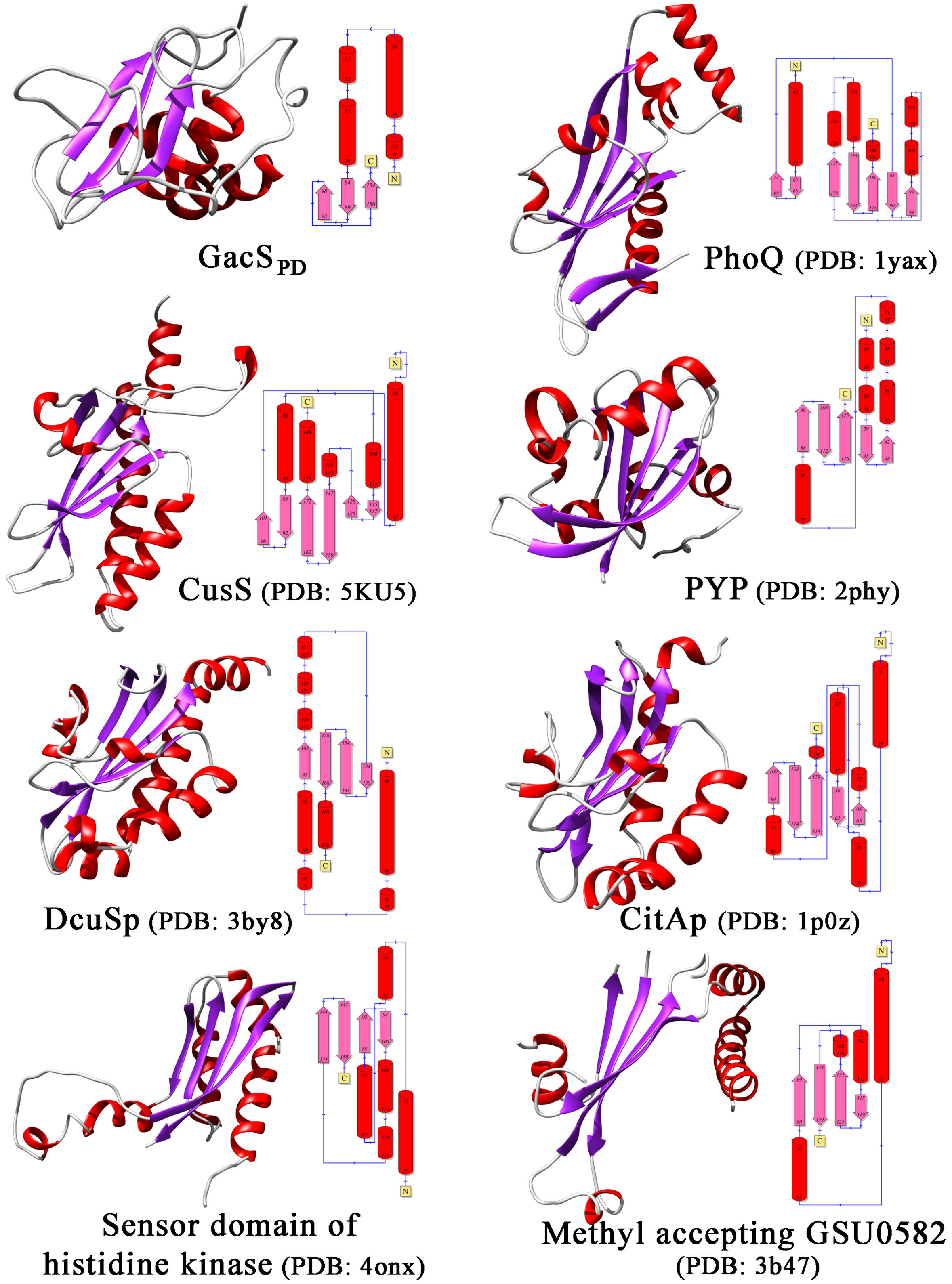

Figure S4: Structural comparison. Overall view of $GacS_{PD}$ (top left) and 7 structural homologues, viewed in a similar orientation, namely the detection domain of PhoQ from *S. typhimurium*, DcuS from *E. coli,* CitA from *K. pneumonia*, CusS from *E. coli*, PYP from *H. halophila,* the sensor domain of Histidine Kinase from *C. perfringens* and the methyl-accepting domain GSU0582 from *G. sulfurreducens.*

Figure S5

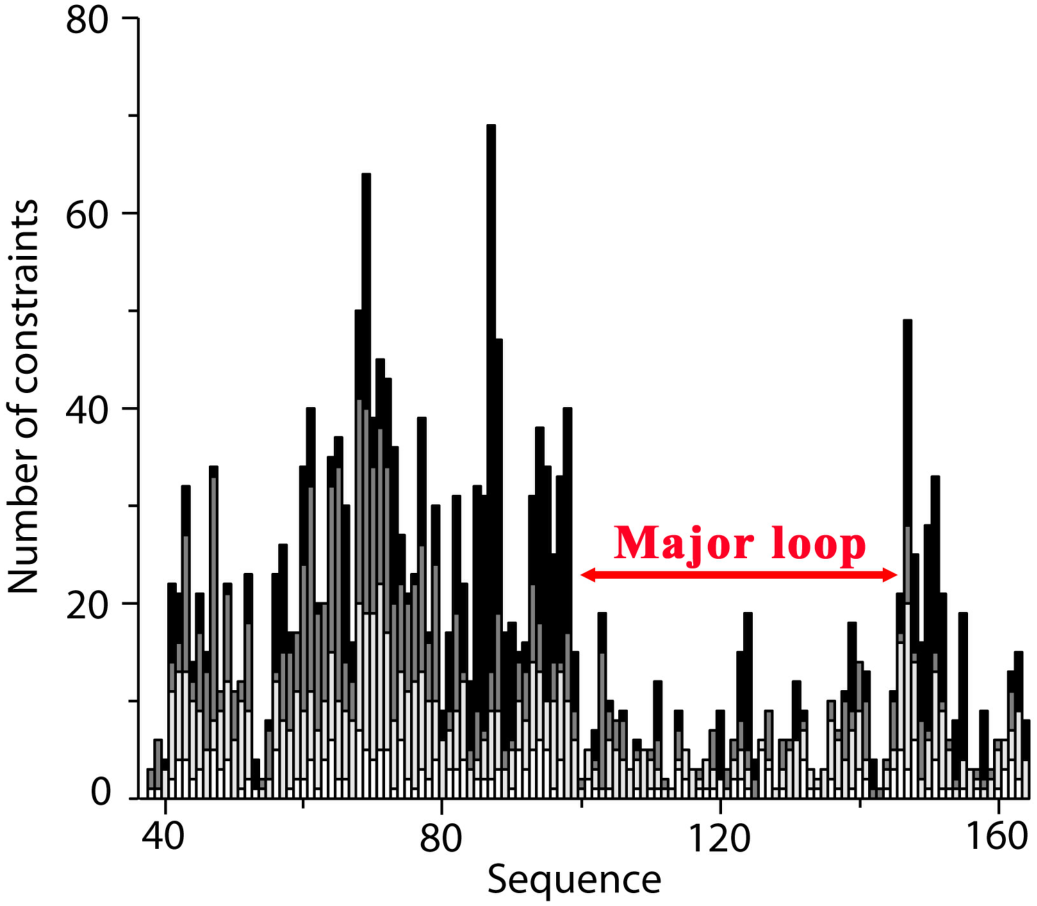

Figure S5: Distribution of the experimental NMR constraints in $GacS_{PD}$. The number of intra-residual (white), short range (light grey), medium-range (dark grey) and long-range (black) restraints is indicated along the amino-acid sequence. The position of the major loop is indicated.

Figure S6

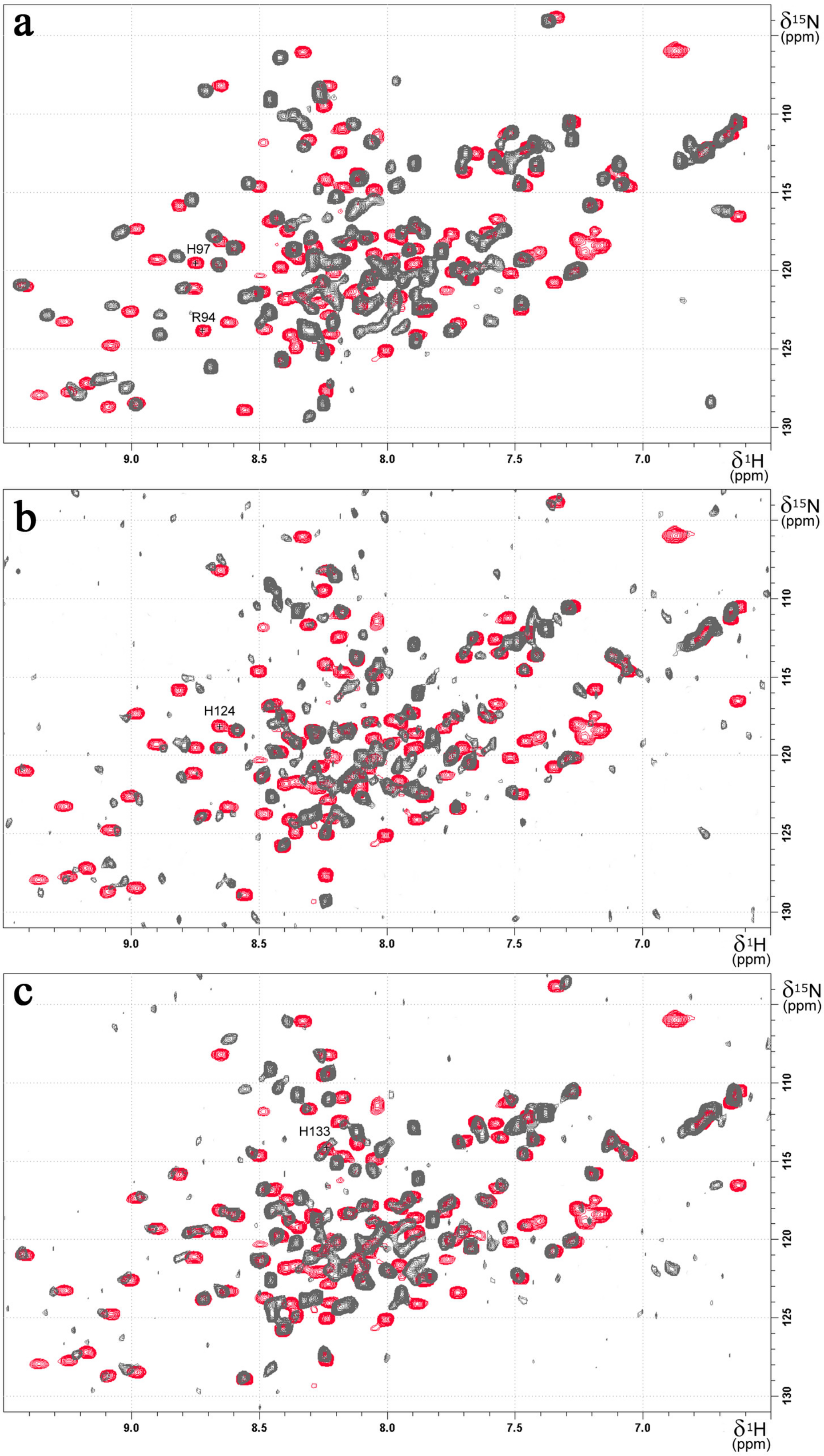

Figure S6: Superimposition of $^{1}H,^{15}N$-HSQC spectra of $GacS_{PD}$ (in red) and $GacS_{PD}$ R94A/H97A (a), H124A (b), H133A (c) (in grey). The samples were prepared in 50 mM potassium phosphate pH 7, 150mM NaCl. The protein concentration of samples was 150 µM. The spectra were recorded at 298K on a 600MHz NMR spectrometer.

Table S5: Molecules used for ligand binding assays with $GacS_{PD}$

Fumarate
Succinate
Pyruvate
Tartrate
Acetate
Citrate
Pyrroloquinoline quinone
Malic acid
L/D-Lactate
Glutamate
N-acetyl Glutamate
Glutamine
Arginine
Homoserine
O-Succinyl-L-homoserine
Putrescine
Spermidine
α-Ketoglutaric acid
Malonic acid
Formate
Glucose
Gluconate
Glutaraldehyde
Reduced/Oxydized Glutathion
Thiamine
Guanosine diphosphate (GDP)
Leucine
γ-aminobutyric acid (GABA)
Fructose
Ions: Ni^{2+}, Mg^{2+}, Fe^{2+}, Zn^{2+}, Mn^{2+}, Ca^{2+}
